# The effect of the VKORC1 promoter variant on warfarin responsiveness in the Saudi WArfarin Pharmacogenetic (SWAP) cohort

**DOI:** 10.1038/s41598-020-68519-9

**Published:** 2020-07-15

**Authors:** Maha Al Ammari, Mohammed AlBalwi, Khizra Sultana, Ibrahim B. Alabdulkareem, Bader Almuzzaini, Nada S. Almakhlafi, Mohammed Aldrees, Jahad Alghamdi

**Affiliations:** 1 Pharmaceutical Care Services, King Abdulaziz Medical City, Ministry of National Guard Health Affairs, King Abdullah International Medical Research Center, King Saud Bin Abdulaziz University for Health Sciences, Riyadh, Saudi Arabia; 2 Department of Pathology and Laboratory, King Abdullah International Medical Research Center, King Saud Bin Abdulaziz University for Health Sciences, Ministry of National Guard Health Affairs, Riyadh, Saudi Arabia; 3 Research Office, King Abdullah International Medical Research Center, King Saud Bin Abdulaziz University for Health Sciences, Ministry of National Guard Health Affairs, Riyadh, Saudi Arabia; 4 Health Sciences Research Center, King Abdullah Bin Abdulaziz University Hospital, Princess Nourah Bint Abdulrahman University, Riyadh, Saudi Arabia; 5 Medical Genomics Research Department, King Abdullah International Medical Research Center, Ministry of National Guard Health Affairs, King Saud Bin Abdulaziz University for Health Sciences, Riyadh, Saudi Arabia; 6 King Abdullah International Medical Research Center, The Saudi Biobank, King Saud Bin Abdulaziz University for Health Sciences, Ministry of National Guard Health Affairs, Riyadh, Saudi Arabia

**Keywords:** Genetics research, Outcomes research

## Abstract

Warfarin is a frequently prescribed oral anticoagulant with a narrow therapeutic index, requiring careful dosing and monitoring. However, patients respond with significant inter-individual variability in terms of the dose and responsiveness of warfarin, attributed to genetic polymorphisms within the genes responsible for the pharmacokinetics and pharmacodynamics of warfarin. Extensive warfarin pharmacogenetic studies have been conducted, including studies resulting in genotype-guided dosing guidelines, but few large scale studies have been conducted with the Saudi population. In this study, we report the study design and baseline characteristics of the Saudi WArfarin Pharmacogenomics (SWAP) cohort, as well as the association of the VKORC1 promoter variants with the warfarin dose and the time to a stable INR. In the 936 Saudi patients recruited in the SWAP study, the minor allele C of rs9923231 was significantly associated with a 8.45 mg higher weekly warfarin dose (p value = 4.0 × 10^–46^), as well as with a significant delay in achieving a stable INR level. The addition of the rs9923231 status to the model, containing all the significant clinical variables, doubled the warfarin dose explained variance to 31%. The SWAP cohort represents a valuable resource for future research with the objective of identifying rare and prevalent genetic variants, which can be incorporated in personalized anticoagulation therapy for the Saudi population.

## Introduction

Warfarin is a frequently prescribed oral anticoagulant agent inhibiting the synthesis of the Vitamin K dependent clotting factors II, VII, IX, X as well as anticoagulation factors protein C and S. It also causes anticoagulation by inhibiting the Vitamin K-epoxide reductase complex, which result in the accumulation of reduced vitamin K2, 3-epoxide and vitamin K hydroquinone^1^. Warfarin is indicated for the treatment or prophylaxis of patients with various disease states, including venous thromboembolic events^2^, atrial fibrillation and flutter^3^, cardiac surgery for valve replacement and orthopedic surgery^4,5^. Warfarin exhibits a narrow therapeutic index and is monitored through the International Normalized Ratio (INR), which measures the ability of the blood to clot. The optimal effect of warfarin, for most of the indications, is when the INR ranges from 2 to 3, except for metallic atrial valve replacement, in which a higher INR value is required^6^.

Several studies reported ethnic differences in the warfarin response and dosage requirements^7–9^. Even for populations with very close ancestry within the Middle East and North Africa (MENA) region, differences in allelic frequencies and their impact on warfarin dosing has been documented^10^. This wide inter-individual variability in the warfarin dose is attributed to several factors, including allelic differences of genetic variants in the warfarin metabolizing enzyme, and environmental factors within each population^11,12^. Genetic variants within cytochrome P450 2C9 (CYP2C9) and the vitamin K-epoxide reductase complex (VKORC1) enzyme explain approximately 50% of the dose variability^13^. Most of the pharmacogenetic guidelines consider variants within two genes, CYP2C9*2, CYP2C9*3, and VKORC1:c.−1639C>T (rs9923231)^14^. The differences in the percentage of variance in the warfarin dose explained by VKORC1 in a population are, to a large degree, due to rs9923231^9^. This variant is significantly associated with the warfarin dose in several populations, including Indians, Chinese, Brazilian, Turkish, Russian, and Emiratis^15–20^. All the studies indicated a significant influence on the warfarin response, accounting for 11–32% of the variability in the warfarin dose^9,15–20^. However, few studies with the MENA populations, and particularly the Saudi population, have been conducted to investigate the pharmacogenetic interaction of warfarin responsiveness. Establishing a large cohort of warfarin users would provide opportunities to identify novel risk alleles for drug responses and prevalent diseases, especially with the high consanguinity rate within the Saudi population^21^. Thus, the aim of this study was to report the cohort profile of the Saudi WArfarin Pharmacogenomics (SWAP), as a large prospective cohort for warfarin pharmacogenomic studies, and to assess the value of this cohort by reporting the association of rs9923231 to warfarin effectiveness.

## Methods

### Study design

This study was a prospective multi-center cohort of patients admitted to the King Abdulaziz Medical City, Ministry of National Guard-Health Affairs (MNGHA), in Riyadh and Jeddah, and King Khalid University Hospital in Riyadh. The study was approved by the Institutional Review Board of King Abdullah International Medical Research Center (KAIMRC) with grant number RC12/163, in accordance with the Helsinki Declaration of 1975. All patients provided written informed consent.

### Study participants

The study enrolled patients from January 2014 to April 2018 from three centers in Riyadh and Jeddah, Saudi Arabia. All patients were Saudi nationals, older than 18 years, and for whom warfarin therapy was initiated or patients using warfarin with an INR 2–3. Patients were excluded if their baseline anticoagulation profile was prolonged (INR more than 1.5), aPTT > 1.5–2 times the normal value, baseline bilirubin more than 2.4 gm/dl, had a mechanical heart valve replacement that requires the INR target to be above 3, receiving chemotherapy, and had a confirmed diagnosis of HIV or hepatitis A, B, C. An EDTA peripheral blood sample was drawn for genetic analysis and the clinical variables were retrieved from the hospital’s electronic records system during the hospital admission. The data included demographic and anthropometric variables such as age, gender, weight, and height. The clinical data included comorbidities, renal, liver, and thyroid functions, drug interactions, hemoglobin levels and the anticoagulation profile. In this study, patients were followed-up for 10 days only, regardless of whether the participant was admitted or an outpatient. All the variables were compiled in an electronic database for subsequent analysis.

### DNA extraction and genotyping assay

All patients signed a written informed consent before peripheral blood samples were collected. Genomic DNA was extracted from the peripheral blood using a Qiagen DNeasy kit (Qiagen, Germany) (spin-column protocol) following the manufacturer’s protocol. In brief, the whole blood sample was lysed using a lysis buffer, the DNA was bonded through a silica binding column and eluted through a similar column using an elution buffer. DNA purity and quantity was assessed using the Nanodrop™ spectrophotometer 8,000 and BR Qubit 3 fluorometer respectively, high quality DNA samples are used for genotyping of rs9923231 (VKOR1C −1639 C>T) using a ready-made fluorescence probe assay (C_30403261_20) by Thermo Fisher Scientific, MA, USA. All the genotyping reactions were done with the TaqMan genotyping master mix (Lot No. 0074819). The genotyping procedure was performed following the standard protocol. In brief, a total volume of 25 µl was added in each well of the 96-well plates, as well as 1.25 µl of genotyping master mix, 1.25 µl of genotyping assay, 9.25 µl of nuclease-free water and 2 µl of 20 ng/µl of DNA. The amplification protocol started with a pre-read stage at 60 °C/30 s followed by a hold stage at 95 °C/10 min; the PCR stage is 95 °C/15 s followed by 60 °C/1 min for 50 cycles. The final stage is a post-read stage at 60 °C/30 s. Reporter dyes for qPCR were detected and analyzed by a real-time qPCR ABI QuantStodio 6 FLEX system with TaqMan Genotyper Software.

### Statistical analysis

Continuous variables are summarized as median with interquartile ranges (IQR), and categorical variables as total number and percentage. The allelic frequency of the rs9923231 summary was done in PLINK to calculate the minor allele frequency (MAF) and Hardy–Weinberg Equilibrium p value^22^. A stepwise regression model was used to select significant non-genetic factors by choosing a p value threshold of 0.25 for entry and 0.1 for exiting the model^23^. Only factors with a significant p value < 0.05 were kept in the non-genetic model. The genetic model included the same variables, plus the rs9923231 genotype as coded by 0, 1, and 2 for the genotypes TT, CT, and CC, respectively. The R^2^ and adjusted R^2^ were calculated for the two models. A linear regression model was used to test for the association of rs9923231 with the weekly average dose after adjusting for age, gender, body mass index and smoking status. The average daily dose was calculated as the average warfarin dose from day 3 to day 10. The Cox regression Hazard model has been adopted by several studies to evaluate the effect of genotypes on the time required to reach a stable INR^24,25^. In this study, a Cox regression model was performed to determine the hazard ratio (HR) for achieving the target INR, and a Kaplan–Meier Survival curve was plotted for the different genotypes and log-rank statistics were computed to test the homogeneity of the estimated survival function for the groups^25^.

## Results

### Baseline characteristics

**Figure 1:**
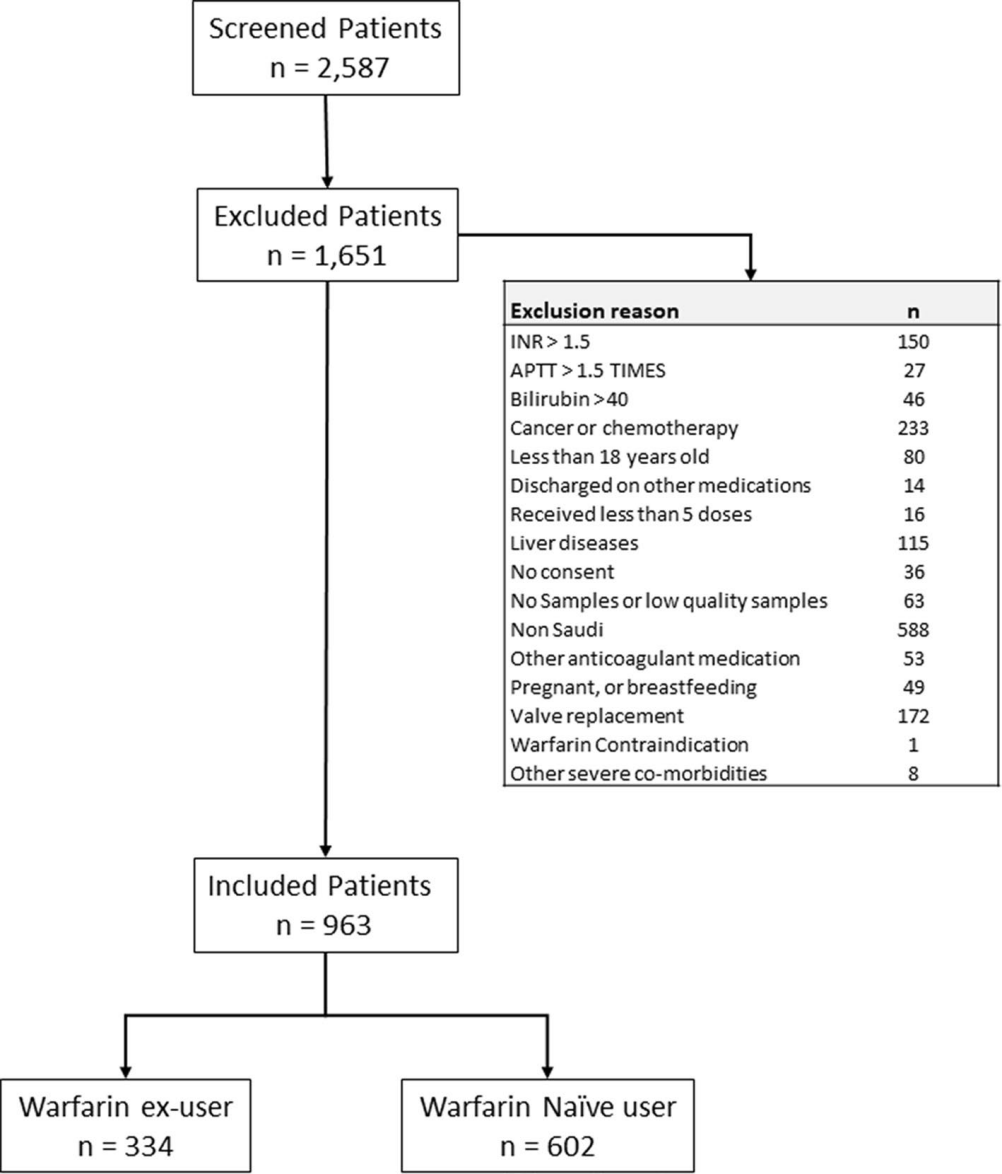
Flow chart of the participants screening step.

During the study recruitment period, a total of 2,581 patients were screened as potential participants in the study (Fig. 1). Of this group, 936 patients fulfilled the inclusion criteria and included in the study. The sample was classified according to the warfarin status on the day of recruitment, to either naïve patients (n = 602), who have never been prescribed warfarin before, or to ex-user (n = 334), who were already on warfarin treatment on the day of recruitment. The baseline characteristics of the sample are displayed in Tables 1 and 2. The average age was 62.4 ± 18.9 years, and 55.2% were female. The most prevalent indications for warfarin were atrial fibrillation (AF, 44.7%), pulmonary embolism (PE, 18.7%), and deep vein thrombosis (DVT, 18.3%). The most frequent comorbidities were hypertension (75%), diabetes (54%), and hyperlipidemia (42%).

**Table 1:** The categorical variables of the baseline characteristics of the patients.

Variables	Category	Ex-user	Naïve	All	Diff
Gender	Male	139 (0.32)	301 (0.68)	440 (0.47)	0.014
	Female	195 (0.39)	301 (0.61)	496 (0.53)	
Smoking*	Yes	9 (0.12)	64 (0.88)	73 (0.08)	**2.32E−06**
	No	309 (0.38)	506 (0.62)	815 (0.92)	
**Indication**					
Atrial fibrillation	Yes	227 (0.54)	191 (0.46)	418 (0.45)	**8.53E−27**
	No	107 (0.21)	409 (0.79)	516 (0.55)	
Deep vein thrombosis	Yes	38 (0.22)	133 (0.78)	171 (0.18)	**2.54E−05**
	No	296 (0.39)	467 (0.61)	763 (0.82)	
Pulmonary embolism	Yes	35 (0.2)	140 (0.8)	175 (0.19)	**5.56E−07**
	No	299 (0.39)	460 (0.61)	759 (0.81)	
Orthopedic surgery	Yes	0 (0)	7 (1)	7 (0.01)	0.0126
	No	334 (0.36)	593 (0.64)	927 (0.99)	
Stroke	Yes	19 (0.29)	46 (0.71)	65 (0.07)	0.248
	No	315 (0.36)	554 (0.64)	869 (0.93)	
Others	Yes	47 (0.26)	131 (0.74)	178 (0.19)	0.00319
	No	287 (0.38)	469 (0.62)	756 (0.81)	
**Co-morbidities**					
Diabetes	Yes	200 (0.39)	308 (0.61)	508 (0.54)	0.0101
	No	134 (0.31)	294 (0.69)	428 (0.46)	
Hypertension	Yes	270 (0.39)	419 (0.61)	689 (0.74)	**1.44E−04**
	No	64 (0.26)	183 (0.74)	247 (0.26)	
Hyperlipidemia	Yes	144 (0.36)	251 (0.64)	395 (0.42)	0.67
	No	190 (0.35)	351 (0.65)	541 (0.58)	
Hyperthyroidism	Yes	3 (0.3)	7 (0.7)	10 (0.01)	0.72
	No	331 (0.36)	595 (0.64)	926 (0.99)	
Hypothyroidism	Yes	50 (0.5)	51 (0.5)	101 (0.11)	0.0025
	No	284 (0.34)	551 (0.66)	835 (0.89)	
None	Yes	41 (0.23)	137 (0.77)	178 (0.19)	5.74E−05
	No	293 (0.39)	465 (0.61)	758 (0.81)	
**Drug interactions**					
Induce CYP2C9	Yes	2 (0.13)	13 (0.87)	15 (0.02)	0.0552
	No	318 (0.35)	581 (0.65)	899 (0.98)	
Inhibit CYP2C9	Yes	31 (0.21)	116 (0.79)	147 (0.16)	6.41E−05
	No	289 (0.38)	478 (0.62)	767 (0.84)	
None	Yes	287 (0.38)	467 (0.62)	754 (0.82)	1.36E−05
	No	33 (0.21)	127 (0.79)	160 (0.18)	
**Renal function**					
GFr, ml/min	More than 50	242 (0.36)	423 (0.64)	665 (0.72)	0.482
	Between 10 and 50	76 (0.37)	132 (0.63)	208 (0.22)	
	Less than 10	16 (0.29)	40 (0.71)	56 (0.06)	
**Stabilized INR**					
Stable INR during 10 days	Yes	212 (0.32)	454 (0.68)	666 (0.71)	**0.0001**
	No	122 (0.45)	148 (0.55)	270 (0.29)	

Bold shows a statistical significant differences between naïve and ex-user group.

**Table 2:** The continuous variables of the baseline characteristics of the patients.

Variable	Ex-user		Naive		All		Diff.^a^
	Mean (SD)	n	Mean (SD)	n	Mean (SD)	n	
Age, years	**67.2 (15.1)**	**334**	**59.6 (20)**	**602**	**62.3 (18.7)**	**936**	**2.60E−09**
BMI, kg/m^2^	**31.4 (8.5)**	**334**	**29.7 (8.9)**	**602**	**30.3 (8.8)**	**936**	**0.0036**
Average daily dose	**3.8 (2)**	**334**	**3.5 (2)**	**602**	**3.6 (2)**	**936**	**0.014**
**Average weekly dose**	**24.55 (0.56)**	**334**	**26.88 (0.76)**	**602**	**25.38 (13.8)**	**936**	**0.014**
rs9923231—CC	**38.63 (1.63)**	**64**	**33.71 (1.10)**	**130**	**35.28 (0.92)**	**194**	**0.05**
rs9923231—CT	26.31 (1.02)	163	24.94 (0.77)	261	25.45 (0.62)	424	0.27
rs9923231—TT	20.12 (1.26)	107	18.07 (0.86)	211	18.74 (0.72)	318	0.09
**Days to stable INR**	**5.99 (4.4)**	**334**	**7.28 (2.8)**	**602**	**6.82 (3.5)**	**936**	**8.83E−08**
rs9923231—CC	**5.68 (4.5)**	**64**	**8.18 (2.8)**	**130**	**7.38 (3.6)**	**194**	**0.000008**
rs9923231—CT	**5.95 (4.4)**	**163**	**7.18 (2.8)**	**261**	**6.72 (3.5)**	**424**	**0.001**
rs9923231—TT	6.19 (4.5)	107	6.82 (2.8)	211	6.62 (3.4)	318	0.13
**Coagulation profile**							
INR (3–10 days)	**2.2 (0.64)**	**334**	**2.27 (0.66)**	**602**	**2.25 (0.65)**	**936**	**0.09**
Prothrombin time, s	**22.4 (11.5)**	**333**	**13.7 (10)**	**602**	**16.8 (11.4)**	**935**	**2.46E−31**
aPTT, ec	**39.7 (14.9)**	**331**	**33 (13.3)**	**602**	**35.4 (14.3)**	**933**	**2.32E−12**
**Liver function test**							
Total bilirubin, μmol/L	12.2 (9.4)	333	12.3 (9.6)	596	12.3 (9.5)	929	0.98
**Albumin, g/L**	**36 (7.8)**	**314**	**33.5 (11)**	**595**	**34.4 (10.1)**	**909**	**0.0003**
ALP, U/L	105.2 (82.8)	333	112 (71.6)	595	109.6 (75.8)	928	0.1862
ALT, U/L	25.6 (33.4)	333	36.8 (119.7)	596	32.8 (98.1)	929	0.0959
AST, U/L	27.7 (31)	334	37.8 (82)	598	34.2 (68.4)	932	0.0316
LDH, U/L	**253.7 (149.7)**	**286**	**291.3 (164.2)**	**539**	**278.2 (160.2)**	**825**	**0.0013**
GTP, U/L	86.4 (120.9)	247	106.1 (195.5)	491	99.5 (174.3)	738	0.1457
**Thyroid function**							
TSH, MIU/L	4.1 (16.5)	318	4.1 (19.2)	551	4.1 (18.3)	869	0.9875
**Complete blood counts (CBC)**							
Hematocrit, L/L	**1.3 (11.3)**	**334**	**3.6 (10.9)**	**602**	**2.8 (11.1)**	**936**	**0.0017**
Hemoglobin, G/L	**119.8 (25.2)**	**334**	**113.5 (35.4)**	**602**	**115.7 (32.3)**	**936**	**0.0043**
RBC, 10^12^/L	6.5 (27.5)	334	7.7 (38.4)	602	7.3 (34.9)	936	0.6125
Platelet, 10^9^/L	266.6 (123.2)	334	284.4 (124)	602	278.1 (124)	936	0.0356
**Renal function test**							
BUN, mmol/L	9.8 (13.1)	334	9.6 (12.2)	602	9.7 (12.5)	936	0.792
Creatinine, μmol/L	138.3 (165.7)	334	136.5 (159.9)	602	137.2 (161.9)	936	0.8728

*aPTT* activated partial thromboplastin time, *ALP* alkaline phosphatase, *ALT* alanine amino transferase, *AST* aspartate amino transferase, *BMI* body mass index, *BUN* blood urea nitrogen, *RBC* red blood cells, *TSH* thyroid stimulating hormone, *LDH* lactate dehydrogenase, *GTP* gamma-glutamyl transferase.

^a^Compare the mean difference for ex-user group to the naïve group by ANOVA.

Bold shows a statistical significant differences between naïve and ex-user group.

### Genotyping rs9923231

**Figure 2:**
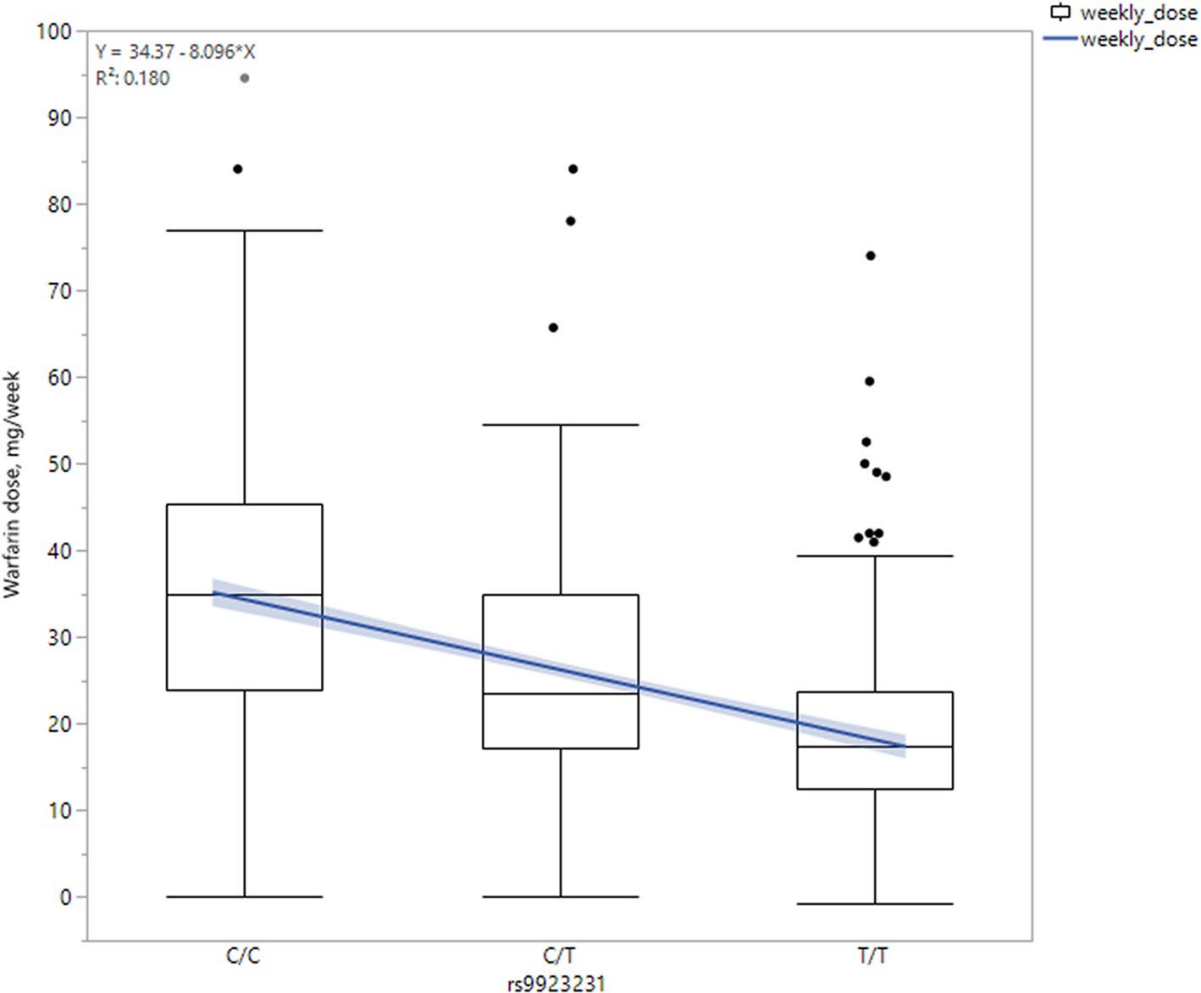
Average of warfarin weekly dose by rs29923231 genotype status.

The minor allele frequency of rs9923231 allele C was 0.43, and the genotypic frequencies were 0.21, 0.45, and 0.34 for genotypes CC, CT, and TT, respectively (Table 3). The genotypic frequency was not significantly different between the groups on initiation or in the maintenance phase (p value = 0.27). The average weekly dose of warfarin was significantly different between the three genotypes (p value = 1.81E-40), with 35.28 mg (95% CI 33.48–37.08), 25.45 mg (95% CI 24.23–26.66), and 18.73 mg (95% CI 17.33–20.14) for the groups of CC, CT, and TT carriers, respectively (Fig. 1). In addition, the rs9923231 genotype was significantly associated with the daily warfarin dose; each copy of the C allele was associated with a 1.16 mg higher dose of warfarin (p value = 3.55 × 10^–45^, Table 3) after adjusting for age, BMI, gender, and smoking status. At a week interval, the warfarin dose was higher by an average of 8.45 mg/week (p value = 4.0 × 10^–46^) for each additional allele-C copy (Fig. 2).

**Table 3:** Genotypic frequency of rs9923231 and association with average weekly dose of warfarin.

Status	CHR	SNP	BP	Minor Allele	Major Allele	Minor Allele Frequency	Genotype frequency			Effect size (mg)	p value*
							CC	CT	TT		
Overall	16	rs9923231	31096368	C	T	0.43	194 (0.21)	424 (0.45)	318 (0.34)	1.17	3.55E−45
Naive						0.43	130 (0.22)	261 (0.43)	211 (0.35)	1.14	6.21E−31
Ex-user**						0.43	64 (0.19)	163 (0.49)	107 (0.32)	1.23	9.37E−16

*Model is adjusted for age, gender, BMI, and smoking status.

**Chi Square test for the difference in genotypic frequencies between the initiation and maintenance groups is not significant (p value = 0.273).

Bold shows a statistical significant differences between naïve and ex-user group.

### Time to stable INR

**Figure 3:**
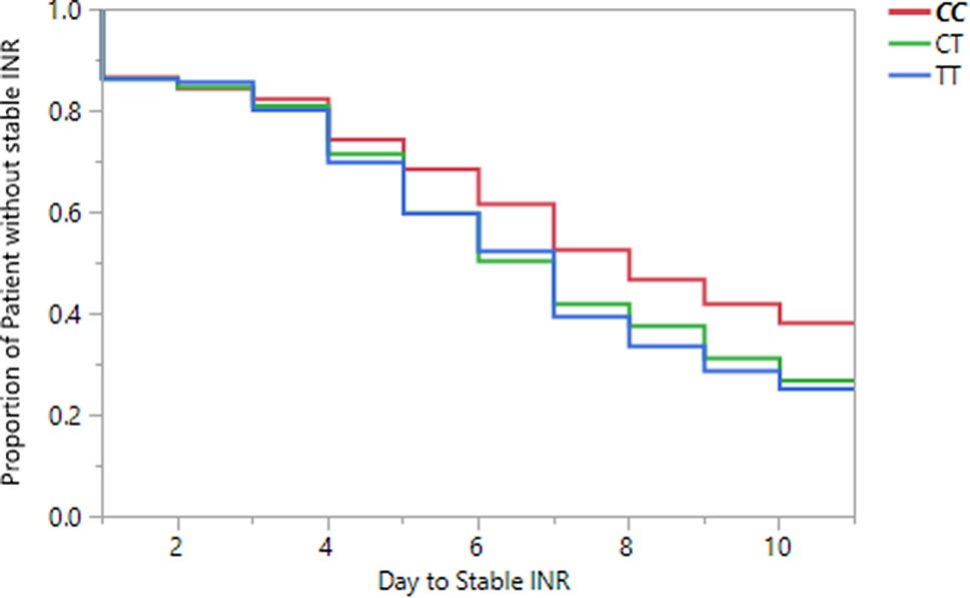
Time to stable INR between the three genotypes.

The Cox regression model indicated a significant association of the rs9923231 genotype with the time to a stable INR (Fig. 2). Patients with the CC genotype had a statistically significant delay in achieving a stable INR, compared to the TT carriers. By day 10, 62% of the CC genotype carriers achieved a stable INR compared to 73% and 75% of the CT and TT carriers, respectively, a statistically significant difference (p value = 0.008, figure). The median time to achieve a stable INR was 8 days for the CC genotype carriers, compared to 7 days for the carriers of the remaining genotypes. The HR of CC carriers for not achieving a stable INR during the first 10 days was 1.33 (95% confidence interval 1.07–1.64, p value = 0.01) compared to the CT carriers and 1.39 (95% CI 1.1–1.71, p value = 0.0052) compared to the TT carriers (Fig. 3).

### Warfarin dose

**Figure 4:**
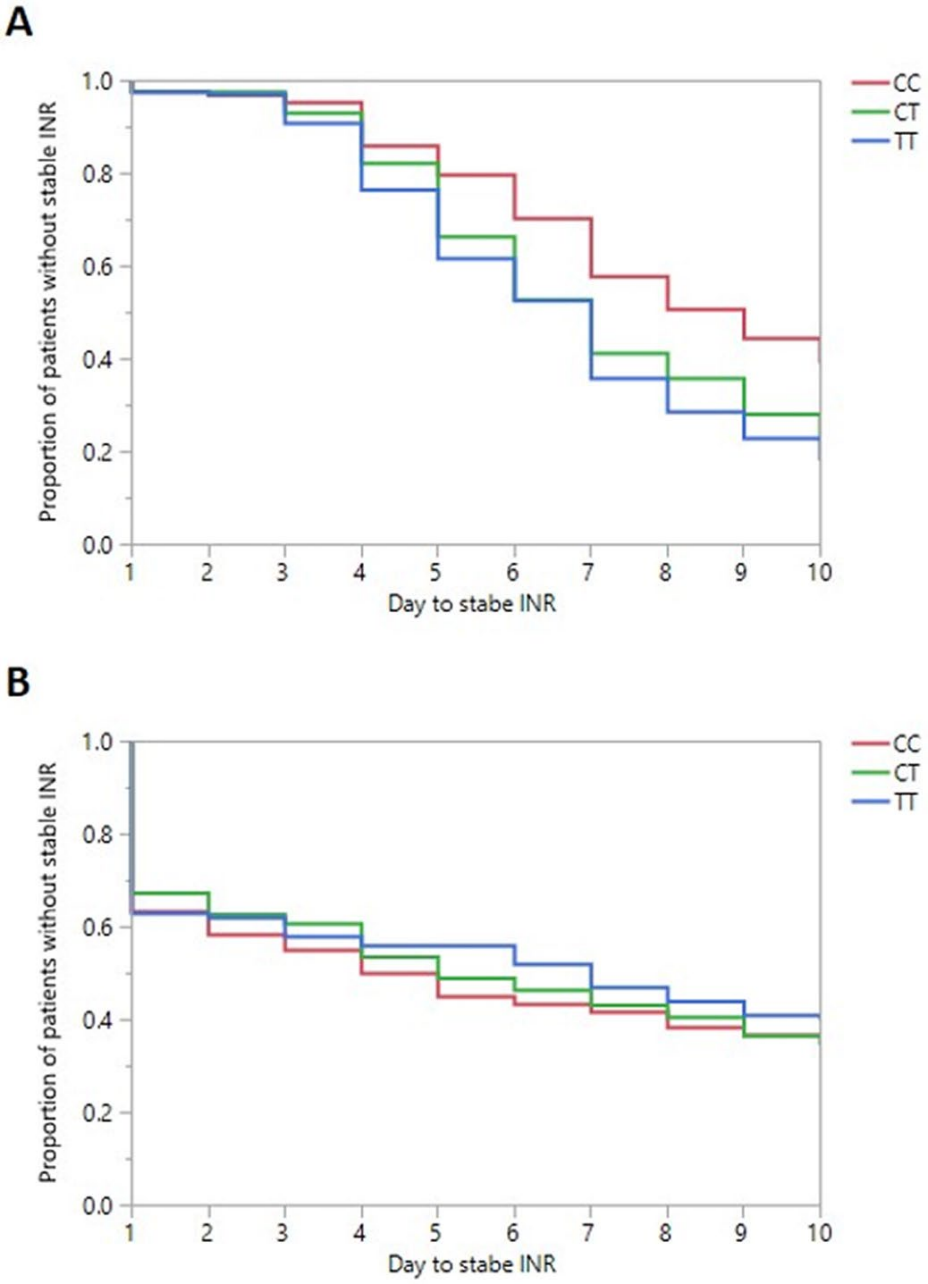
Number of days to reach a stable INR for (**A**) Warfarin Naïve group and (**B**) Warfarin ex-user patients.

In the subgroup of 602 warfarin naïve patients, the C allele of rs9923231 was significantly associated with an average of 1.14 mg higher dose of warfarin (p value = 6.21E−31, Table 3). At day 10, 61% of the CC carriers achieved a stable INR compared to 78% and 82% of the CT and TT carriers, respectively (p value = 0.00003, Fig. 4A). The HR for not achieving a stable INR within the first 10 days of initiating the warfarin dose was significantly higher for the CC carriers by 1.53 (95% confidence interval 1.18–2.00, p value = 0.001) compared to CT, and by 1.76 (95% confidence interval 1.35–2.32, p value = 0.00005) compared to TT, respectively. In the subgroup of 334 maintenance dose patients, the rs9923231-C was also significantly associated with an average of 1.24 mg higher dose of warfarin (p value = 9.37E−16, Table 3). However, there were no statistically significant difference between the three genotypes in maintaining a stable INR during the first 10 days of observation (p value = 0.75, Fig. 4B).

### Non-genetic factors

All non-genetic factors were entered in the stepwise multiple regression model to identify the clinical variables significantly associated with the warfarin dose. The stepwise regression identified seven factors namely, age, weight, status of warfarin, stroke as an indication, the laboratory measurements of red blood cells (RBC), Gamma-Glutamyl Transferase (GTP), and Alkaline phosphatase (Table 4). These factors explained 14% of the daily warfarin dose. The addition of rs9923231 into the model explained an additional 17%, with total of 31% variance explained by the full genetic model (Table 4).

**Table 4:** Stepwise parameters for the impact of non-genetic and genetic models on the warfarin daily dose.

Variable	Non-genetic mode			Genetic model		
	Estimate	Std error	p value	Estimate	Std error	p value
Intercept	4.64	0.40	3.00E−28	3.69	0.38	**3.00E−21**
rs29923231 C				1.09	0.08	**7.00E−35**
Age	− 0.03	0.00	7.00E−13	− 0.03	0.00	**3.00E−15**
Weight	0.02	0.00	8.10E−07	0.02	0.00	**1.30E−07**
Maintenance dose	0.27	0.07	2.20E−04	0.26	0.07	**1.00E−04**
GTP	− 0.001	0.0004	0.01	− 0.001	0.0004	**0.02**
AlkPhos	− 0.002	0.001	0.02	− 0.002	0.001	**0.02**
RBC	0.005	0.002	0.03	0.004	0.002	0.10
Indication stroke [no.]	− 0.25	0.13	0.05	− 0.22	0.11	0.06
R^2^	0.32			0.15		
Adjusted R^2^	0.31			0.14		

## Discussion

The SWAP cohort is the largest cohort of Saudi warfarin using patients with the aim of studying the pharmacogenetics of warfarin responsiveness. In this relatively large cohort, we found a statistically significant association between rs9923231 and the average daily warfarin dose as well as the number of days to achieve a stable INR. The addition of the rs9923231 genotype to the warfarin dose prediction model, that included non-genetic factors, doubled the explained variation to 31%. The rs9923231 was a statistically significant factor that determined the number of days required to achieve a stable INR when initiating warfarin, but this significance was not found with the group receiving a maintenance dose.

The role of genetic variants within VKORC1 is known and have been incorporated in genotype-based dosing algorithms in several populations^13^. Essentially, warfarin antagonizes the Vitamin K-dependent clotting pathway, in which the VKORC1 gene product, VKORC1 protein, is the rate-limiting step in Vitamin K recycling^26^. As the rs9923231 is located in the promoter region of VKORC1, it alters the promoter activity and the transcription factor binding site, leading to a reduction by almost 44% in the luciferase activity of the T allele compared to the C allele^27^. This polymorphism is the most prevalent polymorphism in VKORC1 associated with the warfarin dose and sensitivity^13,28,29^. The International Warfarin Pharmacogenetics Consortium (IWPC) recommend reducing the weekly warfarin dose for carriers of the C allele^23^.

Limited studies investigated the impact of the pharmacogenetic effect on warfarin treatment in Saudi patients. A study with 112 Saudi patients reported an allele frequency estimate of 45% for allele C, similar to the current study^30^. Other studies were conducted with healthy volunteers to estimate the allelic frequency^31,32^. Comparing 499 healthy Saudi participants to 1,105 Europeans, and 106 South Africans, indicated the allele frequencies for the C allele as 0.46, 0.42, and 0.36, respectively^31^. Based on the allele frequencies, this study predicted a statistically significant difference in the warfarin dose between the three populations, with the Saudi population having an average warfarin dose equal to 35.38 mg/week [95% confidence intervals 35.23–36.44, compared to 37.88 mg/week [95% CI 37.41–38.36] and 34.83 mg/week [95% CI 32.41–37.26] for the European and South African populations, respectively.

The IWPC dosing guideline recommends decreasing the average dose of warfarin for patients with the rs9923231 TT and CT genotypes by − 16.14 mg and − 8.97 mg/week, respectively, compared to patients with similar characteristics but a carrier of the CC genotype^23^. In our study, the average of warfarin dose for TT and CT carriers was lowered by − 16.54 and − 9.83 mg/week, respectively. Although these estimates are based solely on the VKORC1 status with no consideration of other important factors such as the CYP2C9 genotype, it indicates that patients treated by the traditional dosing method, have converged to groups with an average dose close to the IWPC dosing guidelines. This does not imply that the full dosing algorithm can be applied in the Saudi population, as other factors could be different between the populations. Other rare variants within VKORC1 and other important pharmacogenes (VIP) could play major roles in populations differences^33^. It must be considered that the clinical value of a genotype-based dosing algorithm, over the standard dosing approach, is still unclear^25,34,35^. To determine whether this result will translate into significant clinical benefits or whether the work describes interesting rare genetic variants remains to be examined.

In this study, we achieved a relatively large sample size of warfarin patients from an under-represented population. The SWAP cohort provides a valuable resource for future studies to develop genotype-based dosing algorithms, targeting the Saudi population. Although we only tested the pharmacogenetic impact of VKORC1 on the warfarin response, the study serves as proof of concept to justify future research to assess the full spectrum of genetic variants using advanced technologies, such as Next-generation Sequencing. One limitation of this study is the fact that we followed-up the patients for 10 days only. During which, it is difficult to draw an informative interpretation about the quality of clinical setting in patients, taking into consideration that the majority of our patients have never been exposed to warfarin at the time of enrollment in the study. Nevertheless, longer follow-up analysis will be conducted in future studies by following the patient electronic health record.

In conclusion, SWAP represents an unprecedented resource as the largest national cohort of warfarin using patients to support a range of studies, with the ultimate goal of identifying rare and prevalent variants to develop personalized anticoagulation treatment. We reported the association of the VKORC1 promoter variant with the warfarin dose and time to a stable INR in the Saudi population. The findings establish a baseline for additional studies to assess the impact of genotype-guided warfarin dosing by using the SWAP materials.
